# Pharmacological management of modifiable cardiovascular risk factors (blood pressure and lipids) following diagnosis of myocardial infarction, stroke and diabetes: comparison between population-based studies in Russia and Norway

**DOI:** 10.1186/s12872-020-01513-1

**Published:** 2020-05-19

**Authors:** Sarah Cook, Laila A. Hopstock, Anne Elise Eggen, Katie Bates, Olena Iakunchykova, Anna Kontsevaya, Martin McKee, Henrik Schirmer, Michael Voevoda, Alexander V. Kudryavtsev, Sofia Malyutina, David A. Leon

**Affiliations:** 1 Department of Community Medicine, UiT The Arctic University of Norway, 9037 Tromsø, Norway; 2 Department of Health Services Research and Policy, London School of Hygiene & Tropical Medicine, 15-17 Tavistock Place, London, WC1H 9SH UK; 3 Department of Medical Statistics, Informatics and Health Economics, Medical University of Innsbruck, Schöpfstraße 41/1, A-6020 Innsbruck, Austria; 4 National research center for preventive medicine, Moscow, Russian Federation 101990; 5 Akershus University Hospital, 1478 Lørenskog, Norway; 6 Institute of Clinical Medicine, University of Oslo, 0318 Oslo, Norway; 7 Research Institute of Internal and Preventive Medicine, Branch of Institute of Cytology and Genetics, Siberian Branch of the Russian Academy of Sciences, Novosibirsk, Russian Federation 630090; 8 Northern State Medical University, Arkhangelsk, Russian Federation 163000; 9 Novosibirsk State Medical University, Russian Ministry of Health, Novosibirsk, Russian Federation 630091; 10 Department of Non-Communicable Disease Epidemiology, London School of Hygiene & Tropical Medicine, Keppel Street, London, WC1E 7HT UK

**Keywords:** Secondary prevention, Russian Federation, Norway, Myocardial infarction, Stroke, Diabetes

## Abstract

**Background:**

Cardiovascular disease (CVD) mortality is substantially higher in Russia than in neighbouring Norway. We aimed to compare blood pressure- and lipid-lowering medication use and proportion meeting treatment targets between general population samples in the two countries in those with CVD and diabetes.

**Methods:**

The study population was adults aged 40–69 years reporting a diagnosis of myocardial infarction (MI), stroke and/or diabetes participating in cross-sectional population-based studies in Russia (Know Your Heart (KYH) 2015–18 *N* = 626) and Norway (The Tromsø Study 2015–16 (Tromsø 7) *N* = 1353). Reported medications were coded according to the 2016 WHO Anatomical Therapeutic Chemical Classification system. Treatment targets were defined using the Joint European Societies guidelines for CVD prevention in clinical practice (2016).

**Results:**

Age- and sex-standardized prevalence of use of lipid-lowering medications was higher in Tromsø 7 for all three conditions with a disproportionately large difference in those reporting MI (+ 48% (95% CI 39, 57%)). Proportion meeting treatment targets for LDL cholesterol was poor in both studies (age- and sex-standardized prevalence of control KYH vs Tromsø 7: MI 5.1% vs 10.1%; stroke 11.6% vs 5.8%; diabetes 24.9% vs 23.3%). Use of antihypertensive medication was higher in KYH for stroke (+ 40% (95% CI 30, 50%)) and diabetes (+ 27% (95% CI 19, 34%)) groups but approximately equal for the MI group (− 1% (95% CI -1, 1%)). Proportion meeting blood pressure targets was lower in KYH vs Tromsø 7 (MI 51.8% vs 76.3%; stroke 49.5% vs 69.6%; diabetes 51.9% vs 63.9%).

**Conclusions:**

We identified different patterns of medication use in people with CVD and diabetes. However despite higher use of lipid-lowering medication in the Norwegian study treatment to target for total cholesterol was poor in both Russian and Norwegian studies. In contrast we found higher levels of use of antihypertensive medications in the Russian study but also that less participants met treatment targets for blood pressure. Further work should investigate what factors are responsible for this seeming paradox and how management of modifiable risk factors for secondary prevention could be improved.

## Background

Cardiovascular disease (CVD) mortality is particularly high in Russia, [1, 2] exceeding that in neighbouring Norway by a factor of four in 2015 [3]. Excess mortality in Russia contributes substantially to the CVD burden in Europe as a whole.

Management of modifiable risk factors such as high blood pressure and lipid levels following CVD diagnosis is crucial for long term prognosis [4, 5]. This includes both lifestyle changes and pharmacological management with appropriate antihypertensive and lipid-lowering medications, and management of co-existing diabetes according to regularly updated European treatment guidelines [5]. Management of blood pressure and lipid levels is also important in patients with diabetes to prevent CVD events.

EUROASPIRE IV [4] (2012–13) collected data from clinical centres in 24 European countries including Russia on management of CVD risk factors following hospitalisation with coronary heart disease. It showed that, although antihypertensive and lipid-lowering medications were widely used, control of these risk factors, particularly lipids, was sub-optimal across Europe. The three Russian centres achieved better control of blood pressure among those taking antihypertensive medication than other European centres, but had lower use of lipid-lowering medications and fewer patients reaching targets for cholesterol reduction [6]. However, it is unlikely that the Russian EUROASPIRE clinical centres, all located within the Moscow oblast, are typical. The EUROSPIRE findings with respect to blood pressure control are inconsistent with data from Russian registries of CVD patients where levels of control were considerably lower [7, 8]. Moreover a multi-centre population survey found that the use of lipid-lowering medication was substantially lower than found in EUROASPIRE IV [9].

In the context of the extremely high levels of cardiovascular disease mortality in Russia we investigate here whether there are differences in secondary prevention (following myocardial infarction and stroke) and prevention in a high risk group (those with diabetes) between Russia and Norway, a neighbouring country with far lower CVD mortality, using population-based samples to capture real world practices.

## Methods

The study population comprised individuals aged 40–69 years with a self-reported diagnosis of myocardial infarction (MI), stroke and/or diabetes and data on medication use who were taking part in the Know Your Heart Study [10] (KYH) conducted in two Russian cities (Arkhangelsk and Novosibirsk (2015–18)) and the seventh wave of the Tromsø Study (Tromsø 7) [11] conducted in the Norwegian city of Tromsø (2015–16). These studies were conducted in parallel as part of the Heart to Heart project aimed at understanding the reasons for very high rates of CVD mortality in Russia through comparisons between the Know Your Heart Study and Tromsø 7 study. Several aspects of data collection between the studies have been harmonized (including the ATC coding of medications, blood pressure measurement protocols and calibration of lipid measurements) providing a unique opportunity to compare the general population of both countries.

### Definition of disease status

As this paper focuses on secondary prevention, participants needed to be aware of their diagnosis (thereby excluding those with silent MIs or undiagnosed diabetes). Therefore primary case definition was self-report of MI, stroke and/or diabetes, defined using the questions shown in Table 1.

**Table 1:** Questions on self-reported morbidity and use of antihypertensive and lipid-lowering medication

	Know Your Heart (Russia)	Tromsø 7 (Norway)
Self-reported morbidity	Have you ever been told by a doctor (been diagnosed) that you have heart attack/myocardial infarction	Do you have, or have you had a heart attack?
	Have you ever been told by a doctor (been diagnosed) that you have stroke	Do you have, or have you had a cerebral stroke/brain haemorrhage?
	Have you ever been told by a doctor (been diagnosed) that you have diabetes	Do you have, or have you had diabetes?
Self-reported use of medication	Do you take your prescribed blood pressure medication every day? (asked only to participants reporting hypertension)	Do you use, or have you used blood pressure lowering drugs?
	Do you take your prescribed cholesterol medication every day? (asked only to participants reporting high cholesterol)	Do you use, or have you used cholesterol lowering drugs?

We tested more specific case definitions for diabetes, restricting the sample to those who reported diabetes *and* medication for diabetes (International WHO Anatomical Therapeutic Chemical (ATC) codes: A10A, A10B). Since this case-definition could exclude some eligible individuals, such as those who control their diabetes with diet, this was only used in a sensitivity analysis of robustness of findings.

A subset of participants in KYH who attended a medical examination were also asked about diagnosis of MI in a second interview including whether they were hospitalised at the time of the event. Sensitivity analyses with a sample restricted to those in KYH reporting disease in both questionnaires and hospitalisation for MI were carried out to test the robustness of findings with a more specific case definition.

### Pharmacological management of blood pressure and lipids

Both studies asked questions about use of antihypertensive and lipid-lowering medication (see Table 1) but there were differences in the procedures used.

In KYH, a baseline interview was administered by a trained interviewer. Participants who reported ever being diagnosed with hypertension were asked a series of questions about prescription and use of antihypertensive medication (Table 1). An equivalent set of questions were asked to those who reported ever having a diagnosis of high cholesterol. At the end of the interview all participants were invited to attend a health check to which they were asked to bring all their medications. Trained interviewers asked about current medication use and recorded the name, dose, indication and frequency of use of medications (up to 7 medications).

In Tromsø 7, self-adminstered questionnares given to all participants included questions about current or previous use of lipid-lowering and antihypertensives medications (Table 1). Next, participants were asked to state the name of all (prescription and non-prescription) medicines they had used regularly during the last 4 weeks (up to 20 medications). The questionnaire was checked by a trained technician at the study site, and participants had to confirm if no medication use was reported.

For both studies, listed medications were coded using the International WHO Anatomical Therapeutic Chemical (ATC) classification system version 2016 [12]. Antihypertensive medication was defined as use of any medications within the ATC classes C02, C03, C07, C08 or C09. Although this classification could include medications prescribed for other indications this was considered appropriate given these medications have a therapeutic effect on blood pressure regardless of indication for treatment. Lipid-lowering medication was defined as use of any medications within the ATC class C10.

Given the focus here on comparing medication use between the studies, the main analyses are based on ATC codes alone. Sensitivity analyses were conducted using a broader definition of report of medication from ATC codes as above *and/or* self-reported use of antihypertensive or lipid-lowering medication (irrespective of whether a relevant medication was identified among the ATC codes) to investigate robustness of findings using a broader definition of medication use.

### Blood pressure and lipid levels

Blood pressure was measured using automatic blood pressure monitors; OMRON 705 IT (OMRON Healthcare) in KYH, and Dinamap (ProCare 300, GE Healthcare) in Tromsø 7. In both studies three measurements were taken separated by 2 min seated rest. In our analyses the mean of second and third readings were used.

Lipid levels were measured using serum blood samples. In KYH participants were instructed not to eat for 4 h prior to the examination. Collection procedures are described in detail elsewhere [10]. LDL cholesterol (LDL-C) was measured using the enzymatic color test using AU 680 Chemistry System Beckman Coulter devices at a central laboratory in Moscow. In Tromsø 7 participants were non-fasting. Blood samples collected in SST tubes were left for 30 min at room temperature, then centrifuged within 1 h for 10 min at 2000 g. Analyses were done within the same day at Department of Laboratory Medicine at the University Hospital of North Norway, Tromsø. LDL-C was measured using homogeneous enzymatic color method using Cobas 8000 Roche devices. Given there were differences in laboratory methods in terms of analytic methods and devices a calibration study was performed comparing measurement of LDL-C in both laboratories on a subset of the same samples. In accordance with the results, KYH LDL-C levels were shifted by using the equation: LDL-C post calibration = − 0.66 + 1.11 (LDL-C pre-calibration).

Targets for control of blood pressure and lipids were taken from the 2016 Joint European Societies guidelines for CVD prevention in clinical practice [5].

The treatment target for blood pressure was defined as < 140 mmHg systolic blood pressure (SBP) and < 90 mmHg diastolic blood pressure (DBP). Treatment guidelines for control of blood pressure in people with diabetes are markedly inconsistent between and within countries. Here, in participants reporting diabetes and a previous MI/stroke (at high risk of CVD), the more stringent target of < 130 mmHg SBP and < 80 mmHg DBP was used for blood pressure.

Meeting treatment targets for lipid levels was defined as LDL-C < 1.8 mmol/L (very high risk) for participants reporting MI, stroke, or diabetes with co-morbid MI/stroke and < 2.6 mmol/L (high risk) for participants with diabetes but no prior MI/stroke [5].

We also investigated prevalence of higher risk factor levels of LDL-C ≥ 4.0 mmol/L, SBP ≥160 mmHg or DBP ≥100 mmHg.

### Covariates

We adjusted for key socio-demographic indicators comparable between the two studies (age, sex and education). Education was coded into three categories (lower, middle and higher) based on the education system within each country. In KYH these groups were lower (incomplete secondary and vocational no secondary), middle (complete secondary, vocational and secondary, specialised secondary) and higher (incomplete higher, higher) education. For Tromsø 7, these were lower (primary) middle (upper secondary) and higher (university/university college) education.

As the standard of medical management of people with prior MI, stroke or diabetes might explain differences between Norway and Russia we used a simple proxy to investigate whether differences were explained by differential health care use: whether participants had visited a doctor in the past 12 months. In KYH, this was any visit to a primary care (district) physician/polyclinic cardiologist/other polyclinic specialist/hospital cardiologist or other hospital doctor. In Tromsø 7, this was any visit to a general practitioner/hospital outpatient clinic or medical specialist other than a general practitioner. Visits to psychiatrists or psychologists were not included given our focus on community CVD treatment.

### Analysis

First in descriptive analyses we compared patterns of medication use by study for each condition in terms of 1) prevalence of use of antihypertensive and lipid-lowering medication and 2) prevalence of blood pressure and lipids controlled to treatment guidelines. Prevalences were directly standardised by age and sex to the European 2013 standard population. Then separate multivariable logistic regression models were fitted for each outcome, with incremental adjustments for a) age and sex b) education, and c) visiting a doctor in the past 12 months. The exposure in each model was study using KYH as the reference group.

All analyses were conducted using Stata version 15 [13].

## Results

The characteristics of the participants by condition and study are shown in Table 2. The proportion of participants reporting at least two of the three conditions was higher in KYH than in Tromsø 7. The proportion of participants who visited a doctor in the past 12 months was high in KYH (> 80% for all conditions) but even higher in Tromsø 7 (> 90% for all conditions).

**Table 2:** Characteristics of participants with myocardial infarction, stroke and diabetes in Know Your Heart (KYH) and Tromsø 7

		Myocardial Infarction				Stroke				Diabetes			
		KYH		Tromsø 7		KYH		Tromsø 7		KYH		Tromsø 7	
		N	(%)	N	(%)	N	(%)	N	(%)	N	(%)	N	(%)
Total		240	(100)	376	(100)	144	(100)	309	(100)	341	(100)	792	(100)
Age	40–49	17	(7.1)	31	(8.2)	9	(6.3)	47	(15.2)	34	(10.0)	185	(23.4)
	50–59	66	(27.5)	106	(28.2)	43	(29.9)	92	(29.8)	83	(24.3)	253	(31.9)
	60–69	157	(65.4)	239	(63.6)	93	(63.9)	170	(55.0)	224	(65.7)	354	(44.7)
Sex	Female	101	(42.1)	62	(16.5)	70	(48.6)	127	(41.1)	222	(65.1)	368	(46.5)
	Male	139	(57.9)	314	(83.5)	74	(51.4)	182	(58.9)	119	(34.9)	424	(53.5)
Educational level	Lower	27	(11.3)	114	(31.1)	15	(10.4)	82	(27.3)	28	(8.2)	220	(28.2)
	Middle	143	(59.6)	121	(33.0)	85	(59.0)	102	(34.0)	205	(60.1)	248	(31.8)
	Higher	70	(29.2)	132	(36.0)	44	(30.6)	116	(38.7)	108	(31.7)	312	(40.0)
	Missing	0		9		0		9		0		12	
Visited Doctor in past 12 months	Yes	193	(80.4)	95.1	(95.1)	121	(84.0)	281	(91.8)	300	(88.0)	750	(95.4)
	Missing	0		7		0		3		0		6	
Smoking status	Never	90	(37.8)	79	(21.4)	63	(43.8)	79	(26.2)	179	(52.5)	276	(35.6)
	Ex-smoker	71	(29.8)	218	(58.9)	43	(29.9)	165	(54.6)	99	(29.0)	379	(48.8)
	Current smoker	77	(32.4)	73	(19.7)	38	(26.4)	58	(19.2)	63	(18.5)	121	(15.6)
	Missing	2		6		0		7		0		16	
Co-morbid MI	Yes	–	–	–	–	39	(27.1)	31	(11.0)	40	(11.8)	61	(8.2)
	Missing	–	–	–	–	0		26		1		47	
Co-morbid stroke	Yes	39	(16.3)	31	(8.8)	–	–	–	–	32	(9.4)	39	(5.3)
	Missing	0		22		–	–	–	–	0		50	
Co-morbid diabetes	Yes	40	(16.7)	61	(17.2)	32	(22.2)	39	(13.5)	–	–	–	–
	Missing	0		21		0		21		–	–	–	–
Any Co morbidity^a^	Yes	67	(27.9)	85	(22.6)	59	(41.0)	63	(20.4)	60	(17.6)	93	(11.7)
Mean Blood pressure (95% CI)^c^	SBP	136.6	(133.2, 139.9)	125.6	(122.5, 128.6)	136.7	(133.3, 140.1)	127.8	(125.5, 130.1)	137.0	(133.9, 140.1)	131.6	(130.4, 132.8)
	DBP	85.2	(83.1, 87.3)	75.1	(73.4, 76.8)	84.3	(82.7, 85.8)	76.2	(74.8, 77.6)	85.0	(83.3, 86.8)	76.4	(75.8, 77.1)
	Missing	1		1		3		2		2		3	
Mean Cholesterol Levels (95% CI)^b^,^c^	Total cholesterol	5.3	(5.1, 5.4)	4.5	(4.3, 4.7))	5.2	(5.0, 5.4)	5.0	(4.9, 5.2)	5.1	(4.9, 5.3)	5.1	(5.0, 5.2)
	LDL-C	3.4	(3.3, 3.6)	2.8	(2.6, 3.0)	3.3	(3.1, 3.5)	3.2	(3.0, 3.3)	3.2	(3.1, 3.4)	3.3	(3.2, 3.3)
	Missing	4		3		1		2		7		6	
Mean HbA1c (95% CI)^b, c^		6.0	(5.9, 6.2)	6.0	(5.9, 6.2)	6.1	(6.0, 6.3)	5.9	(5.7, 6.0)	7.4	(7.1, 7.6)	7.3	(7.2. 7.4)
	Missing	5		8		3		5		7		14	
Mean Body mass index (95% CI)^c^		30.2	(6.4)	29.1	(4.3)	29.6	(6.0)	27.7	(4.2)	32.8	(6.2)	30.2	(5.2)
	Missing	1		1		1		5		3		4	

^a^Two or more conditions of MI, stroke or diabetes

^b^ KYH values calibrated to account for differences in laboratory measurements between studies

^c^ Mean values standardized for age and sex to 2013 European standard population

### Use of lipid-lowering and antihypertensive medications

**Fig. 1:**
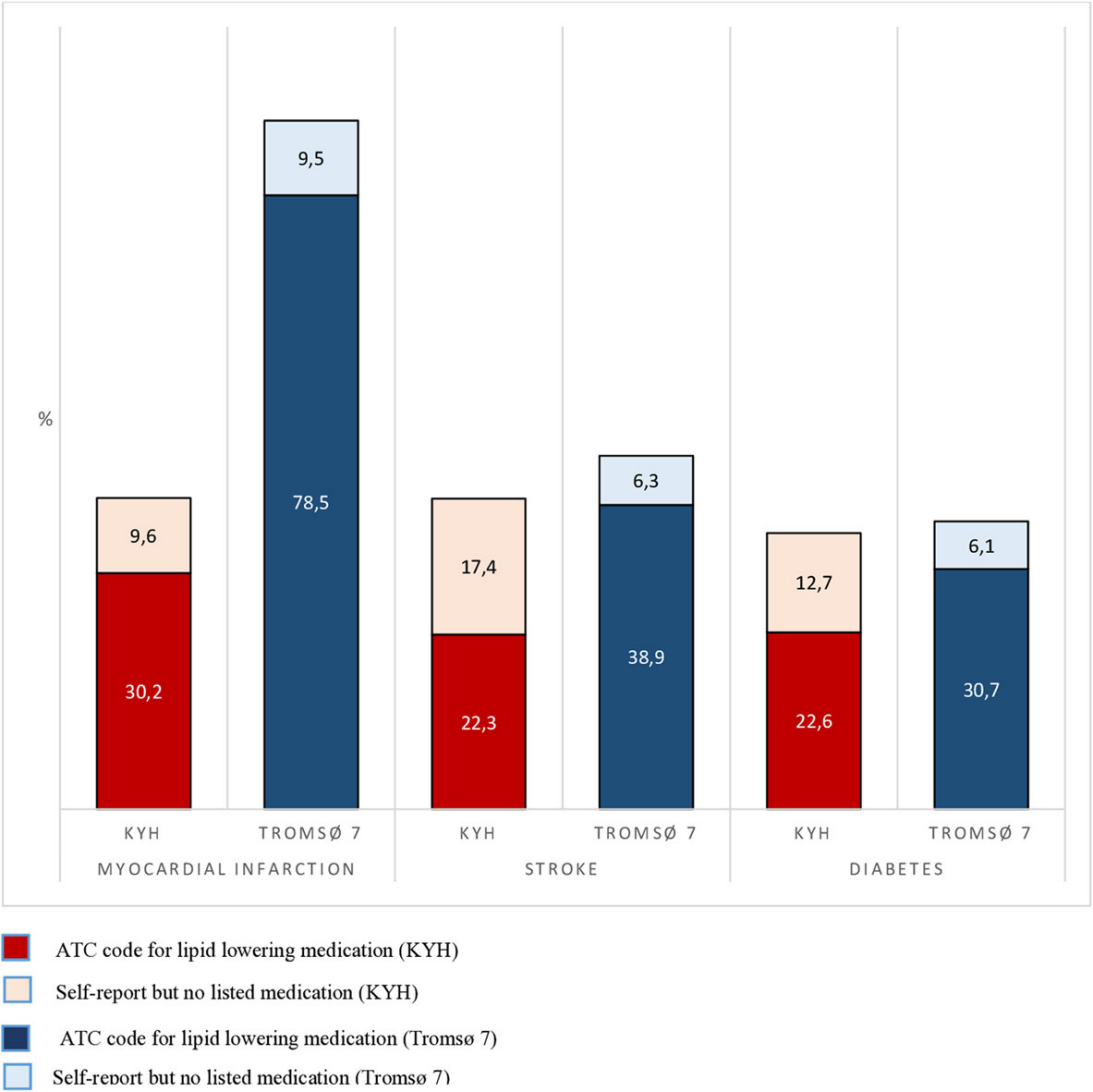
Age- and sex-standardised prevalence of the use of lipid-lowering medications in KYH and Tromsø 7 in participants reporting MI, stroke or diabetes

**Fig. 2:**
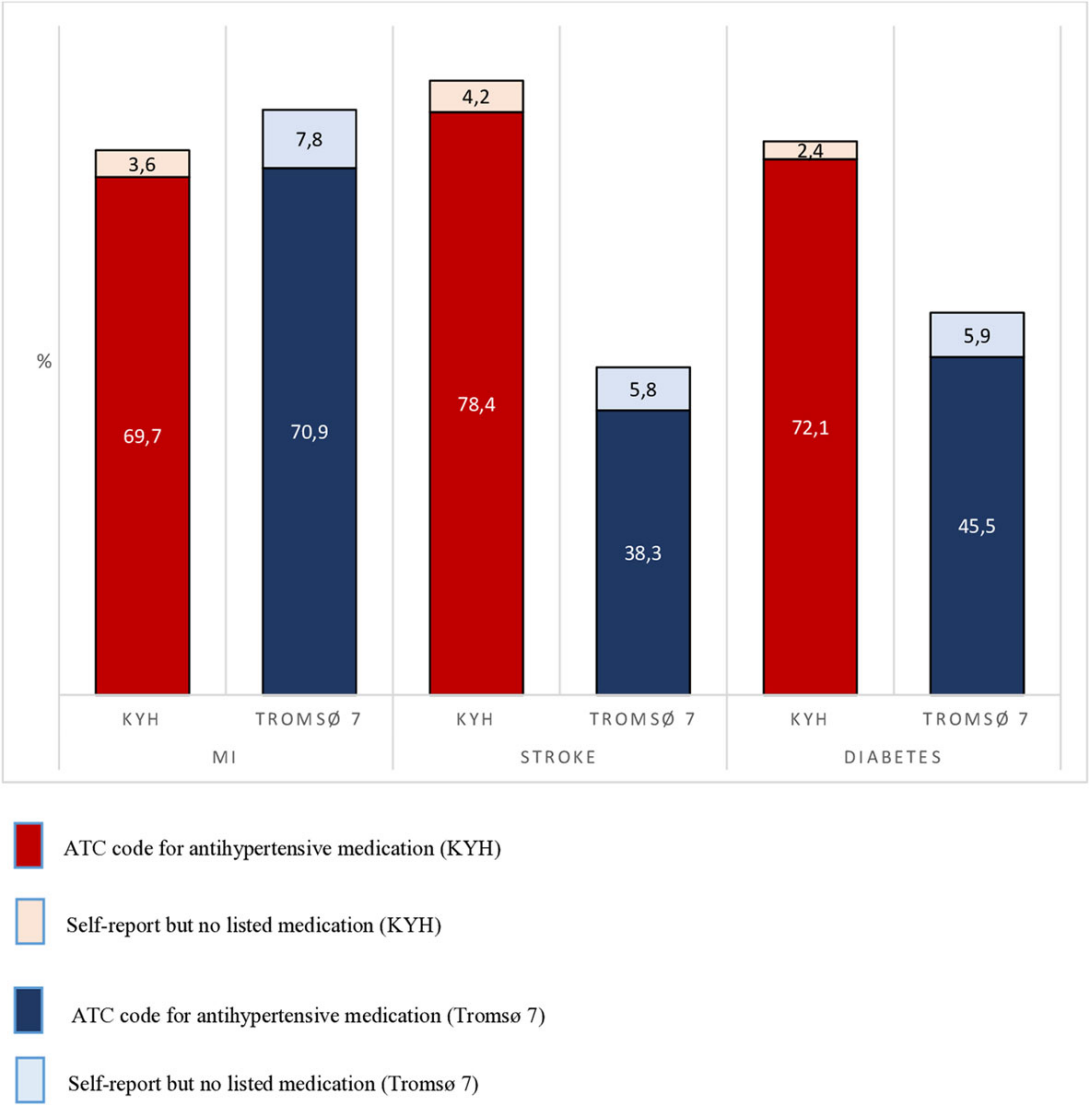
Age- and sex-standardised prevalence of the use of antihypertensive medications in KYH and Tromsø 7 in participants reporting MI, stroke or diabetes

The age and sex-standardised prevalence of use of lipid-lowering and antihypertensive medications by study are shown in Figs. 1 and 2. Prevalences are shown for both ATC codes and for self-reported use but with no specific medication listed.

Using the ATC code definition, use of lipid-lowering medications was higher in Tromsø 7 for all three conditions but with an especially large difference in those reporting MI. Use of medications differed markedly by disease category in Tromsø 7, with much higher proportion of those with MI using lipid-lowering medications than those with stroke or diabetes. This striking difference was not seen in KYH. When broadening the definition of medication use to include those with self-reported use but no ATC code the large between study differences among participants with MI remained, but the differences for stroke and diabetes narrowed. This was due to a higher proportion of participants in KYH reporting use of lipid-lowering medication but with no medication listed.

The prevalence of antihypertensive medication use was similar in KYH and Tromsø 7 for those with MI, but substantially higher in KYH than Tromsø 7 for those with stroke and diabetes. These findings were robust to the definition of medication use.

The between study differences in medication use remained after adjustment for education and visiting a doctor in the past 12 months but with some attenuation of odds ratios for lipid-lowering medication after adjustment for visiting a doctor (Tables 3 and 4).

**Table 3:** Differences in use of lipid-lowering medication and meeting treatment targets for lipids by study

		KYH		Tromsø 7		OR^c^ (Tromsø 7/KYH) adjusted for age and sex (95% CI)	OR^c^ (Tromsø 7/KYH) adjusted for age, sex and education (95% CI)	OR^c^ (Tromsø 7/KYH) adjusted for age, sex, education and visiting a doctor in the past 12 months (95% CI)
		n/total N	Age and sex standardised %^a^	n/total N	Age and sex standardised %^a^			
MI	Use of lipid-lowering medication	91/236	30.2	270/373	78.5	4.23 (2.94, 6.08)	4.34 (2.97, 6.36)	3.94 (2.67, 5.81)
	Meets treatment target for lipid levels^b^	36/236	5.1	41/332	10.1	1.52 (0.80,2.88)	1.33 (0.68, 2.58)	1.29 (0.65, 2.53)
	Meets treatment target for lipid levels^b^ and on medication	27/91	10.9	32/270	10.9	0.87 (0.41, 1.84)	0.77 (0.35, 1.67)	0.75 (0.34, 1.64)
	LDL-C ≥ 4 mmol/L	48/236	24.2	43/373	10.0	0.44 (0.27, 0.69)	0.44 (0.27, 0.72)	0.47 (0.29, 0.77)
Stroke	Use of lipid-lowering medication	43/143	22.3	144/307	38.9	2.31 (1.49, 3.58)	2.20 (1.40, 3.47)	2.08 (1.31, 3.28)
	Meets treatment target for lipid levels^b^	25/143	11.6	21/307	5.8	0.61 (0.30, 1.28)	0.62 (0.29, 1.33)	0.60 (0.28, 1.28)
	Meets treatment target for lipid levels^b^and on medication	21/43	41.9	17/144	9.2	0.25 (0.10, 0.59)	0.25 (0.10, 0.65)	0.25 (0.10, 0.65)
	LDL-C ≥ 4 mmol/L	29/143	29.7	48/307	19.9	0.24 (0.15, 0.39)	0.20 (0.12, 0.34)	0.20 (0.12, 0.34)
Diabetes	Use of lipid-lowering medication	88/336	22.6	272/786	30.7	1.78 (1.32, 2.40)	1.71 (1.25, 2.34)	1.66 (1.21, 2.28)
	Meets treatment target for lipid levels^b^	118/336	24.9	191/786	23.3	1.01 (0.74, 1.39)	1.01 (0.73, 1.40)	1.00 (0.72, 1.40)
	Meets treatment target for lipid levels^b^ and on medication	56/88	33.3	112/272	42.7	0.83 (0.50, 1.37)	0.85 (0.50, 1.44)	0.86 (0.51, 1.45)
	LDL-C ≥ 4 mmol/L	64/336	21.5	182/786	23.7	0.46 (0.28, 0.75)	0.40 (0.23, 0.68)	0.40 (0.23, 0.68)

^a^ Denominator is participants with included data on LDL-C (LDL-C missing for 9 participants in Know Your Heart; 10 participants Tromsø 7)

^b^Treatment targets LDL-C < 1. 8 mmol/L for MI/stroke and diabetes with co-morbid stroke/ MI and < 2.6 mmol/L for diabetes with no co-morbid stroke/MI

^c^Excluding those with missing data on education or visiting a doctor in the past 12 months

**Table 4:** Differences in use of antihypertensive medications and meeting treatment targets for blood pressure by study

		KYH		Tromsø 7		OR^c^ (Tromsø 7/KYH) adjusted for age and sex (95% CI)	OR^c^ (Tromsø 7/KYH) adjusted for age, sex and education (95% CI)	OR^c^ (Tromsø 7/KYH) adjusted for age, sex, education and visiting a doctor in the past 12 months (95% CI)
		n/total N	age and sex standardized %^a^	n/total N	age and sex standardized %^a^			
MI	Use of antihypertensive medication	193/239	69.7	252/375	70.9	0.56 (0.37, 0.84)	0.56 (0.37, 0.86)	0.51 (0.33, 0.79)
	Meets treatment target for blood pressure^b^	114/239	51.8	255/375	76.3	2.44 (1.71, 3.48)	2.74 (1.89, 3.99)	2.60 (1.77, 3.82)
	Meets treatment target for blood pressure^b^and on medication	90/193	38.7	168/252	77.5	2.34 (1.56, 3.52)	2.77 (1.80, 4.28)	2.71 (1.74, 4.21)
	SBP ≥160 mmHg or DBP ≥100 mmHg	43/239	15.9	26/375	6.0	0.35 (0.20, 0.59)	0.33 (0.19, 0.58)	0.37 (0.21, 0.65)
Stroke	Use of antihypertensive medication	115/141	78.4	147/308	38.3	0.21 (0.13,0.34)	0.20 (0.12, 0.34)	0.18 (0.11, 0.31)
	Meets treatment target for blood pressure^b^	62/141	49.5	207/308	69.6	2.62 (1.72, 3.99)	2.66 (1.71, 4.13)	2.68 (1.72, 4.18)
	Meets treatment target for blood pressure^b^ and on medication	44/115	38.1	95/147	74.1	3.08 (1.83, 5.18)	3.48 (1.99, 6.08)	3.47 (1.98, 6.10)
	SBP ≥160 mmHg or DBP ≥100 mmHg	30/141	12.8	12/308	2.6	0.16 (0.08, 0.32)	0.15 (0.07, 0.32)	0.16 (0.08, 0.32)
Diabetes	Use of antihypertensive medication	277/339	72.2	387/790	45.5	0.25 (0.18, 0.34)	0.26 (0.19, 0.36)	0.24 (0.17, 0.34)
	Meets treatment target for blood pressure^b^	171/339	51.9	479/790	63.9	1.42 (1.08, 1.86)	1.39 (1.05, 1.85)	1.34 (1.01, 1.79)
	Meets treatment target for blood pressure^b^ and on medication	138/277	53.5	217/387	57.9	1.28 (0.93, 1.78)	1.28 (0.90, 1.80)	1.25 (0.88, 1.77)
	SBP ≥160 mmHg or DBP ≥100 mmHg	67/339	17.6	48/790	5.2	0.29 (0.19, 0.44)	0.29 (0.19, 0.44)	0.31 (0.20, 0.48)

^a^ Denominator is participants with data on measured blood pressure (missing for 5 participants Know Your Heart; 5 participants Tromsø 7)

^b^Treatment target for blood pressure SBP < 140 mmHg and DBP < 90 mmHg for MI/stroke and no co-morbid diabetes/ diabetes with no comorbid stroke/MI or SBP < 130 mmHg and DBP < 80 mmHg for diabetes with co-morbid stroke or MI/ stroke with comorbid diabetes

^c^Excluding those with missing data on education or visiting a doctor in the past 12 months

### Between study differences in meeting treatment guidelines for lipids and blood pressure

The proportion meeting treatment targets for lipid levels in each study is shown in Table 3. The majority of participants in each study had LDL-C levels which did not meet treatment targets. Proportion meeting treatment targets for LDL-C did not differ between the two studies in individuals with any of the conditions either in the total population or among those on medication. The exception to this was those with stroke where among those on medication a higher proportion met treatment targets in the KYH group, however it should be noted this sub-group only included 43 participants. The prevalence of LDL-C ≥ 4.0 mmol/L was higher in KYH than Tromsø 7 for all three conditions with the largest magnitude of effect in those with MI or stroke (Table 3).

Proportion meeting treatment targets for blood pressure in each study is shown in Table 4. Despite higher use of antihypertensive medication in KYH, more participants met the treatment guidelines in Tromsø 7 for all conditions. For MI and stroke, the odds ratios (comparing Tromsø 7 and KYH) were similar (2–3 times higher) whether considering all participants or when restricted to those on medication. Among those with diabetes, the odd ratios were smaller and confidence intervals crossed 1 when restricting analyses to those on medication. The prevalence of SBP ≥160 mmHg or DBP ≥100 mmHg was higher in KYH than Tromsø 7 for all conditions.

Adjustment for education and visiting a doctor did not explain the between study differences.

### Sensitivity analyses with more specific case definition

Sensitivity analyses showed that more specific case definitions of diabetes for KYH and Tromsø 7 (Supplementary Table 1) and MI for KYH (Supplementary Table 2) made no material difference.

## Discussion

In this paper we have compared the pharmacological management of modifiable CVD risk factors between participants reporting MI, stroke, and diabetes in two population-based studies conducted in Russia and Norway.

The use of lipid-lowering medications was higher in Tromsø 7, with a particularly large difference among those with a history of MI. Reduction of LDL-C to the guidelines was very low in both countries (age and sex standardized prevalence of control: MI 5.1% vs 10.1% stroke 11.6% vs 5.8% diabetes 24.9% vs 23.3%). Conversely the use of antihypertensive medications was lower in Tromsø 7. Despite this, the proportion meeting treatment targets for blood pressure was lower in KYH than Tromsø 7 for all conditions (age and sex standardized prevalence of control: MI 51.8% vs 76.3% stroke 49.5% vs 69.6% diabetes 72.2% vs 45.5%).

It is important that the very high levels of use of antihypertensive medications in KYH were not reflected in lower blood pressures. Further investigation is need to explain this but it may reflect, in part, differences in how people obtain medication in Russia compared to Norway. In Russia antihypertensive and lipid-lowering medications can be obtained from pharmacies without a prescription so high levels of medication use do not necessarily reflect equivalent levels of monitoring by a health professional. Also, medication use was assessed by participants’ reports of what medications they were using and we have not assessed adherence to medication. Intermittent use of medication (for example only when feeling unwell) could be a factor in poor levels of control. There may be other reasons for poor blood pressure control in Russia, such as a co-morbid chronic kidney disease, which need to be investigated.

Here we have investigated pharmacological management in a general population setting reflecting what actually happens in practice. Our findings can be contrasted with EUROASPIRE IV [4] which took place in a controlled clinical setting. EUROASPIRE IV participants were hospital patients where the quality of care may plausibly be higher than in the general population. Compared to EUROASPIRE IV the age- and sex-standardised prevalence of use of antihypertensive medications (78.1%) was similar in the MI participants using the broader definition of medication use in both Tromsø 7(73.3%) and KYH (78.7%). With this definition use of lipid-lowering medications was also similar in EUROASPIRE IV (86.6%) to Tromsø 7 (88.0%) while in KYH even with the broad definition of medication use this was only 39.8%. This is lower than the Russian EUROASPIRE IV centres where 74.6% of participants were taking statins [6]. This discrepancy could be an indicator of regional variation in secondary prevention of CVD within Russia. The EUROASPIRE centres were all within the Moscow oblast and included specialized clinical centres where adherence to treatment guidelines may be higher than found throughout the whole country. In a population survey in 13 regions of the Russian Federation (2012–2013) only 40.0% of males and 28.1% of females with history of MI were taking statins (for stroke patients these numbers were 13.9 and 15.7%, for diabetes 12.2 and 12.7%) [9]. However our finding that the target of LDL-C < 1.8 mmol/L was not met in the majority of participants even in the Norwegian MI group is consistent with EUROASPIRE IV (80.5% ≥1.8 mmol/L) reflecting both the difficulty in achieving this target and a general need for more intensive lipid management in high risk groups throughout Europe.

This study has some limitations: firstly the number of participants in each disease group was small. This was reduced further when restricting to those taking medication. Despite low power, there was strong evidence of large between study differences in medication use and proportion meeting treatment guidelines that are unlikely to be explained by chance.

Secondly, definitions of disease status and medication use were self-reported and subject to potential misclassification, with some differences in methodology in the two studies. Literature on reporting of self-reported medication use compared to electronic registry data suggests high validity for CVD medications [14–16]. Here we examined the impact of different definitions of medication use and found the substantive findings remained the same. Literature on the validity of self-reported CVD morbidities, including validation of self-reported stroke in a previous wave of the Tromsø Study [17] suggests that these are specific but not sensitive, with lower sensitivity for stroke and diabetes than MI [17–21]. While lower sensitivity of self-report means we may have missed some participants eligible for inclusion this is unlikely to have affected the results unless the people excluded differed substantially in terms of medication use and control from those included in the two studies and under-reporting of disease status differed by study. The sensitivity analyses with more specific case definitions found the results were robust, although it should be noted that they had even lower power than the main study due to the smaller sample size when restricting to those identified with a stricter case definition.

Here we did not have the capacity within the data to determine stroke sub-types. There is debate about the use of statins following haemorrhagic stroke [22, 23], therefore different proportions of stroke sub-type within the studies could have impacted on subsequent medical management with lipid-lowering medication. However the consistent finding of differences across all three conditions suggests we have identified a fundamental difference in pharmacological management. Similarly we did not have data on contraindications for medication use (statin intolerance, hyperkalemia), however unless there are large differences in the populations in prevalence of contraindications this is very unlikely to have affected the study results.

Finally, participants were recruited from two cities in Russia and one in Norway so the findings cannot be considered representative of the whole of each country.

## Conclusions

In conclusion we have found that among people with a self-reported history of MI there was a lower level of use of lipid-lowering medications in the Russian compared to Norwegian study. In contrast we found higher levels of use of antihypertensive medications in the Russian study but also that less participants met treatment targets for blood pressure. Treatment to target for total cholesterol was poor in both Russian and Norwegian studies. Further work should investigate what factors are responsible for this seeming paradox and how management of modifiable risk factors for secondary prevention could be improved.

## Supplementary information

**Additional file 1: Table S1.** Sensitivity analysis of findings with more specific case definition of diabetes. **Table S2.** Sensitivity analysis of findings with more specific case definition of MI for KYH study

## Data Availability

The data that support the findings of this study are available from Know Your Heart and The Tromsø Study, but restrictions apply to the availability of these data, which were used under license for the current study, and so are not publicly available. Data from the Know Your Heart Study are however available from the authors upon reasonable request with permission of Know Your Heart [24]. For The Tromsø Study, data are available subject to scientific and ethical approval of a study protocol [25].

## Abbreviations

ATC code: WHO international anatomical therapeutic chemical code
CVD: Cardiovascular disease
DBP: Diastolic blood pressure
KYH: Know your heart
LDL-C: Low density lipoprotein cholesterol
MI: Myocardial infarction
SBP: Systolic blood pressure
