# Supplementary material for: Pharmacological management of modifiable cardiovascular risk factors (blood pressure and lipids) following diagnosis of myocardial infarction, stroke and diabetes: comparison between population-based studies in Russia and Norway
Source: BMC Cardiovasc Disord. 2020 May 19;20:234. doi: 10.1186/s12872-020-01513-1 (PMC7236339; doi:10.1186/s12872-020-01513-1)
Supplement: Supplementary file 1 — Additional file 1: Table S1. Sensitivity analysis of findings with more specific case definition of diabetes. Table S2. Sensitivity analysis of findings with more specific case definition of MI for KYH study [file 12872_2020_1513_MOESM1_ESM.docx]

**Supplementary Table 1: Sensitivity analysis of findings with more specific case definition of diabetes**

|  | | Age and sex standardised prevalence (%) | | Age and sex adjusted odds ratio (Tromsø 7/KYH) 95% CI |
| --- | --- | --- | --- | --- |
|  |  | KYH | Tromsø 7 |  |
| Use of lipid-lowering medication (medication list) | Self-reported | 22.6 | 30.7 | 1.78 (1.32, 2.40) |
|  | Self-report + Use of medication for diabetes | 25.8 | 39.9 | 2.25 (1.58, 3.21) |
| Meets target guidelines for LDL-C^a^ | Self-reported | 24.9 | 23.3 | 0.99 (0.72, 1.35) |
|  | Self-report + Use of medication for diabetes | 27.1 | 27.2 | 0.86 (0.59, 1.26) |
| Meets target guidelines for LDL-C^a^ among those on medication | Self-reported | 33.3 | 42.7 | 1.20 (0.73, 1.98) |
|  | Self-report + Use of medication for diabetes | 27.3 | 44.1 | 1.04 (0.59, 1.84) |
| LDL-C ≥4 mmol/L | Self-reported | 21.5 | 23.7 | 0.69 (0.28, 1.66) |
|  | Self-report + Use of medication for diabetes | 18.9 | 19.3 | 0.64 (0.23, 1.78) |
| Use of antihypertensive medication (medication list) | Self-reported | 72.1 | 45.5 | 0.25 (0.18, 0.34) |
|  | Self-report + Use of medication for diabetes | 77.6 | 56.0 | 0.25 (0.16, 0.39) |
| Meets target guidelines for blood pressure^b^ | Self-reported | 51.9 | 63.9 | 1.42 (1.08, 1.86) |
|  | Self-report + Use of medication for diabetes | 55.0 | 64.1 | 1.26 (0.90, 1.76) |
| Meets target guidelines for blood pressure^b^  among those on antihypertensive medication | Self-reported | 53.5 | 57.9 | 1.28 (0.93, 1.78) |
|  | Self-report + Use of medication for diabetes | 54.5 | 59.0 | 1.19 (0.81, 1.74) |
| SBP ≥160 mm Hg or DBP ≥100 mm Hg | Self-reported | 17.6 | 5.2 | 0.30 (0.19. 0.44) |
|  | Self-report + Use of medication for diabetes | 10.4 | 4.8 | 0.44 (0.25, 0.77) |

For lipid-lowering medication use analyse restricted to those with measured LDL-C and for antihypertensive medication use analyses restricted to those with measured blood pressure

N with diabetes self-report and use of medication for diabetes with data on LDL-C (KYH=231 Tromsø 7=511) and with measured blood pressure (KYH=232 Tromsø 7=514). On lipid-lowering medication KYH=70 Tromsø 7=230 On Antihypertensive medication KYH=202 Tromsø 7=307

^a^Treatment target for lipids LDL-C <1. 8mmol/L for MI/stroke and diabetes with co-morbid stroke/ MI and <2.6mmol/L for diabetes with no co-morbid stroke/MI

^b^Treatment target for blood pressure defined as SBP <140 mm Hg and DBP <90 mm Hg for MI/stroke and no co-morbid/diabetes with no comorbid stroke/MI or SBP <130 mm Hg and DBP <80 mm Hg for diabetes with co-morbid stroke or MI/ stroke or MI with comorbid diabetes

**Supplementary Table 2. Sensitivity analysis of findings with more specific case definition of MI for KYH study**

|  | | Age and sex standardized prevalence (%) | | Age and sex adjusted odds ratio (Tromsø 7/KYH) 95% CI |
| --- | --- | --- | --- | --- |
|  |  | KYH | Tromsø 7 |  |
| Use of lipid-lowering medication (medication list) | Self-reported | 30.2 | 78.5 | 4.23 (2.94, 6.08) |
|  | Self-report at two time points and hospitalisation (KYH)* | 37.4 | 78.5 | 2.78 (1.78, 4.34) |
| Meets target guidelines for LDL-C^a^ | Self-reported | 5.1 | 10.1 | 0.66 (0.35, 1.25) |
|  | Self-report at two time points and hospitalisation (KYH)* | 4.6 | 10.1 | 0.61 (0.26, 1.41) |
| Meets target guidelines for LDL-C^a^ among those on medication | Self-reported | 10.9 | 10.9 | 1.15 (0.54, 2.43) |
|  | Self-report at two time points and hospitalisation (KYH)* | 8.2 | 10.9 | 1.17 (0.48, 2.83) |
| LDL-C ≥4 mmol/L | Self-reported | 24.2 | 10.0 | 0.45 (0.19, 1.08) |
|  | Self-report at two time points and hospitalisation (KYH)* | 16.0 | 10.0 | 0.52 (0.18, 1.52) |
| Use of antihypertensive medication (medication list) | Self-reported | 69.7 | 70.9 | 0.56 (0.37, 0.84) |
|  | Self-report at two time points and hospitalisation (KYH)* | 71.7 | 70.9 | 0.38 (0.21, 0.68) |
| Meets target guidelines for blood pressure^b^ | Self-reported | 51.8 | 76.3 | 2.44 (1.71, 3.48) |
|  | Self-report at two time points and hospitalisation (KYH)* | 33.3 | 76.3 | 2.85 (1.83, 4.45) |
| Meets target guidelines for blood pressure^b^  among those on antihypertensive medication | Self-reported | 38.7 | 77.5 | 2.34 (1.56, 3.52) |
|  | Self-report at two time points and hospitalisation (KYH)* | 30.6 | 77.5 | 2.69 (1.64, 4.43) |
| SBP ≥160 mm Hg or DBP ≥100 mm Hg | Self-reported | 21.4 | 6.0 | 0.35 (0.20,0.59) |
|  | Self-report at two time points and hospitalisation (KYH)* | 21.4 | 6.0 | 0.39 (0.20, 0.73) |

* Tromsø 7 prevalence does not change

N reporting MI at two time points + hospitalisation=108 (2 missing data on LDL) On lipid-lowering medication n=29 On Antihypertensive medication n=92

^a^Treatment target for LDL-C <1. 8mmol/L for MI/stroke and diabetes with co-morbid stroke/ MI and <2.6mmol/L for diabetes with no co-morbid stroke/MI

^b^Treatment target for blood pressure defined as SBP<140 mm Hg and DBP<90 mm Hg for MI/stroke and no co-morbid/diabetes with no comorbid stroke/MI or SBP <130 mm Hg and DBP <80 mm Hg for diabetes with co-morbid stroke or MI/ stroke or MI with comorbid diabetes
